# Decoding illusory self-location from activity in the human hippocampus

**DOI:** 10.3389/fnhum.2015.00412

**Published:** 2015-07-15

**Authors:** Arvid Guterstam, Malin Björnsdotter, Loretxu Bergouignan, Giovanni Gentile, Tie-Qiang Li, H. Henrik Ehrsson

**Affiliations:** ^1^Department of Neuroscience, Karolinska Institutet, StockholmSweden; ^2^Department of Clinical and Experimental Medicine, Linköping University, LinköpingSweden; ^3^Basque Center on Cognition, Brain and Language, DonostiaSpain; ^4^Division of the Humanities and Social Sciences, California Institute of Technology, Pasadena, CAUSA; ^5^Department of Medical Physics, Karolinska University Hospital Huddinge, StockholmSweden

**Keywords:** body perception, perceptual illusion, self-consciousness, self-location, multisensory integration

## Abstract

Decades of research have demonstrated a role for the hippocampus in spatial navigation and episodic and spatial memory. However, empirical evidence linking hippocampal activity to the perceptual experience of being physically located at a particular place in the environment is lacking. In this study, we used a multisensory out-of-body illusion to perceptually ‘teleport’ six healthy participants between two different locations in the scanner room during high-resolution functional magnetic resonance imaging (fMRI). The participants were fitted with MRI-compatible head-mounted displays that changed their first-person visual perspective to that of a pair of cameras placed in one of two corners of the scanner room. To elicit the illusion of being physically located in this position, we delivered synchronous visuo-tactile stimulation in the form of an object moving toward the cameras coupled with touches applied to the participant’s chest. Asynchronous visuo-tactile stimulation did not induce the illusion and served as a control condition. We found that illusory self-location could be successfully decoded from patterns of activity in the hippocampus in all of the participants in the synchronous (*P* < 0.05) but not in the asynchronous condition (*P* > 0.05). At the group-level, the decoding accuracy was significantly higher in the synchronous than in the asynchronous condition (*P* = 0.012). These findings associate hippocampal activity with the perceived location of the bodily self in space, which suggests that the human hippocampus is involved not only in spatial navigation and memory but also in the construction of our sense of bodily self-location.

## Introduction

An extensive history of neurophysiological studies in rats has demonstrated that the hippocampus is crucial for the construction of internal representations of the spatial environment (O’Keefe and Nadel, 1978; Andersen, 2007; Moser et al., 2008). An illustrative example is the seminal discovery of “place cells” (O’Keefe and Dostrovsky, 1971), which described single hippocampal neurons that fire when a freely moving animal occupies a specific location in the local environment. In humans, the hippocampus plays a central role not only in episodic and spatial memory (O’Keefe and Nadel, 1978; Burgess et al., 2002) but also in spatial navigation (Maguire et al., 1998; Hassabis et al., 2009; Howard et al., 2014) and spatial mental imagery (Lambrey et al., 2012; Marchette et al., 2014). However, the involvement of the human hippocampus in spatial perception is less understood, and it remains unknown whether hippocampal activity reflects the perceptual experience of being physically located at a particular place in the environment.

**FIGURE 1:**
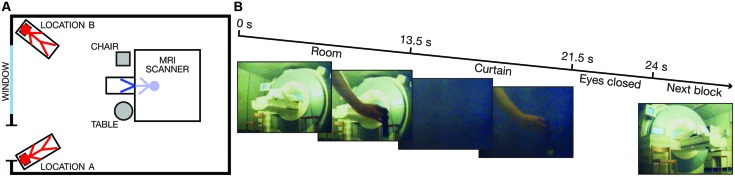
**Scanner room environment and visual stimulus. (A)** A schematic drawing of the scanner room environment. The key spatial landmarks and the illusory self-locations (Locations A and B) are indicated. The red stick figures represent the two illusory self-locations, whereas the blue stick figure indicates the veridical location of the participant’s body inside the bore of the scanner. **(B)** The timing and five representative frames from the 3D visual stimuli (only the image from the left eye is shown) for one experimental block. The first and the last frame indicate the viewpoint from Location A and Location B, respectively.

To address this question, we adapted a perceptual out-of-body illusion (Ehrsson, 2007; Guterstam and Ehrsson, 2012) to the environment of an MRI scanner. Six participants were instructed to lie in a supine position on the scanner bed with their heads tilted forward and wearing head-mounted displays (HMDs). Through the HMDs, the volunteers observed the scanner room in stereoscopic vision from the perspective of a person lying on a bed on the floor in one of two different corners of the room (Locations A and B) and looking at the front of the MRI scanner from these viewpoints (**Figure 1A**). To induce the illusion of being physically located at one of these two locations, the experimenter simultaneously touched the participant’s real chest, which was out of view, and the chest of the ‘illusory body,’ which was located in the corner of the room, just below the field of view (FOV) of the HMDs (**Figure 1B**). After inducing this illusion, a dark curtain was rapidly lowered, and the entire visual field was covered for 8 s (**Figure 1B**) while the experimenter continued touching the ‘illusory body’ to maintain the illusion. In half of the trials, the touching of the real chest and the ‘illusory chest’ occurred asynchronously, which is a mode of visuo-tactile stimulation that significantly reduces the illusion and allows for the comparison of otherwise equivalent conditions (Ehrsson, 2007; Guterstam and Ehrsson, 2012).

**FIGURE 2:**
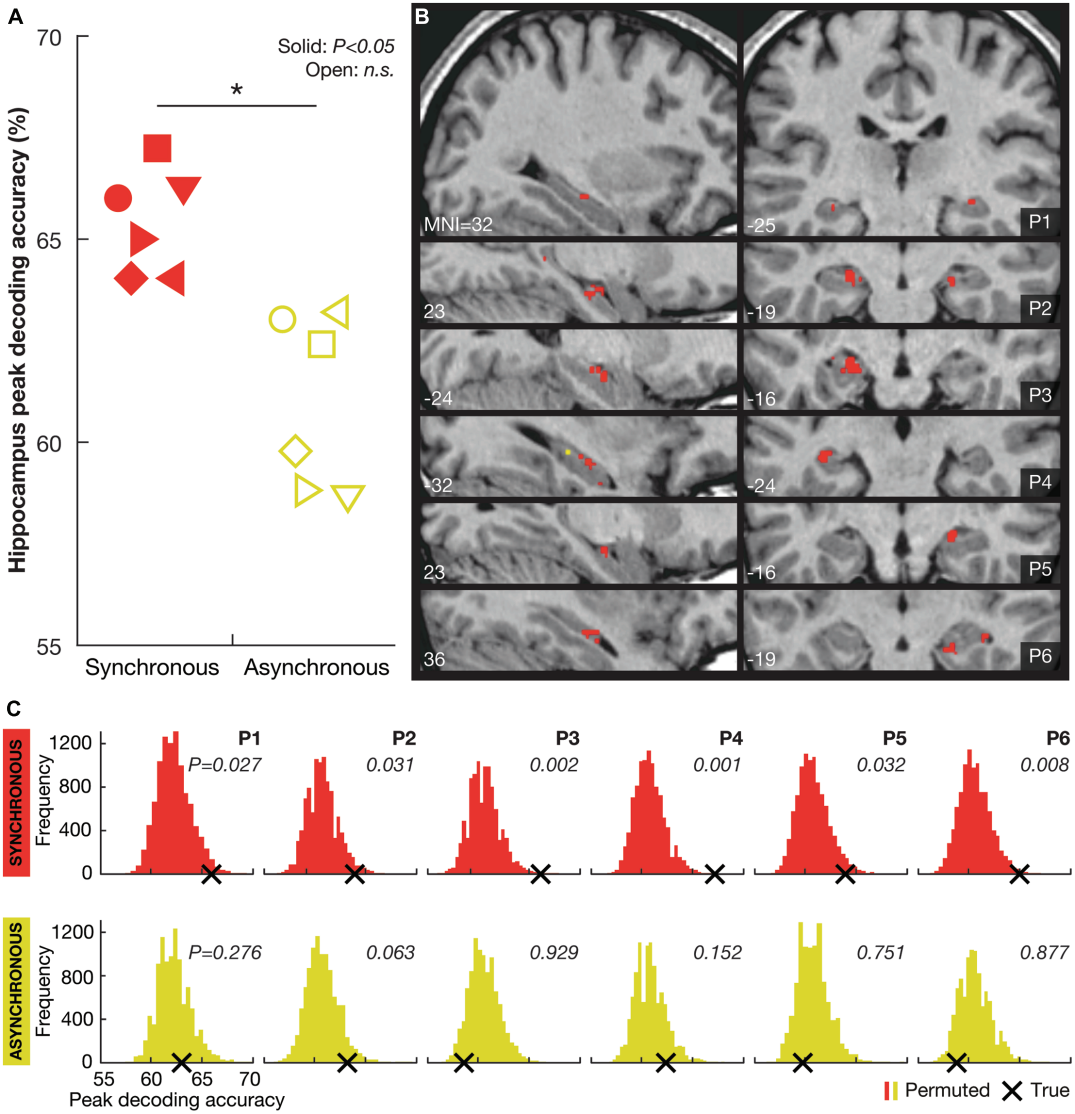
**Decoding results. (A)** Individual peak decoding accuracies for the synchronous (illusion; red symbols) and the asynchronous (control; yellow symbols) conditions. The symbols indicate each participant (female participants are represented by triangles). A solid symbol represents a significant value (*P* < 0.05, corrected) and an open symbol represents a non-significant (n.s.) value (*P* > 0.05, corrected). At the group-level, the peak decoding accuracy was significantly higher in the synchronous than in the asynchronous condition (*P = 0.012). **(B)** Maps illustrating the localization of voxels informative about self-location during the synchronous (red) and asynchronous (yellow) conditions (*P* < 0.001, uncorrected) for each participant (P1–P6). Note that there were significantly fewer (or no) such voxels in the asynchronous condition (mean number of voxels ± SD: 2.0 ± 2.9) than in the synchronous condition (mean number of voxels ± SD: 32.3 ± 15.4; *P* < 0.05, two-tailed Wilcoxon signed-rank test). At the group-level, there were no significant differences among the hippocampal subregions (right anterior, right posterior, left anterior, left posterior; χ^2^(3) = 1.316, *P* = 0.725, Freidman test) in the number of informative voxels. **(C)** Permutation testing. The histograms indicate the participantwise distributions of peak decoding accuracies under the null hypothesis that there was no information regarding LOCATION A and LOCATION B encoded in the hippocampus for the synchronous (red) and asynchronous (yellow) conditions (permutation testing with 10,000 iterations). The frequency of permuted (randomly labeled) decoding maps (*Y* axis) is presented as a function of the peak decoding accuracy (*X* axis). The true peak decoding accuracy is indicated with a black cross (corresponding to the symbols shown in **A**). The probability of obtaining the true value under the null hypothesis (i.e., the *P*-value) is displayed for the synchronous and asynchronous conditions for each participant.

During the course of the experiment, we perceptually ‘teleported’ the participants between Locations A and B multiple times while acquiring high-resolution functional magnetic resonance imaging (fMRI) data. To test the hypothesis that hippocampal activity reflects perceived self-location, we used multivoxel pattern analysis (MVPA) to examine if it is possible to decode illusory self-location from spatially distributed patterns of activity across voxels in the hippocampus. The asynchronous condition and the 8-second-period featuring the dark curtain served as controls for visual input. The results showed that Location A vs. B could be significantly decoded in all six participants in the synchronous illusion condition (**Figure 2**). However, the decoding performance in the asynchronous control condition was not significantly better than chance. These findings associate hippocampal activity with the perceived location of the bodily self in the environment.

## Materials and Methods

### Participants

Six healthy volunteers (mean age ± SD: 28 ± 5.1 years; three females) took part in the experiment. The participants were placed comfortably on the MRI scanner bed in a supine position with their head tilted approximately 25°. Their feet and legs, protruding from the bore of the MRI scanner, were covered with a thin white cloth. Written informed consent was obtained prior to participation, and the Regional Ethical Review Board of Stockholm approved all of the procedures. The participants had no task and were instructed to lie still and look into the HMDs.

### Spatial Environment

The MRI scanner room was 7 m × 5 m with a height of 3 m. A schematic drawing of the room is shown in **Figure 1A**. Before scanning, the participants walked around and explored the room for a few minutes to familiarize themselves with the spatial environment.

### Visual Stimuli

The visual stimuli were presented in the form of stereoscopic (three-dimensional; 3D) videos recorded in the scanner room in a separate session. We used two MRI-compatible cameras (MRC Systems, Heidelberg, Germany) mounted in parallel and 9 cm apart on a custom-made wooden tripod. The cameras were positioned to capture the visual perspective of a person lying on the bed (in Location A or B) with the head tilted approximately 25°, observing the front of the MRI scanner (**Figure 1**). A pair of legs, covered with a white cloth, visibly protruded from the bore of the scanner. The view included the landmarks indicated in **Figure 1A**. All of the spatial aspects of the room were carefully maintained between the video recording session and the subsequent experiment.

Two short 3D videos were recorded for the experiment: one video for Location A and one video for Location B. The duration of each video was 24 s, divided into three different phases (**Figure 1B**). During the first 13.5 s (the ‘*Room*’ phase), the room was fully visible. The experimenter constantly moved his hand, holding a small rod, toward the space just below the FOV as if he were touching the chest of an ‘illusory body’ located in this position. The stimuli included single touches and double touches (two touches delivered in rapid succession with an interval of 300 ms) applied at irregular intervals (average frequency: 0.46 Hz) according to a pre-determined sequence provided to the experimenter through audio cues transmitted via MRI-compatible headphones. The visual stimuli were either temporally congruent or incongruent with the tactile stimulation of the participant’s chest (see Tactile stimuli below). The touches of the ‘illusory body’ were applied at identical positions and distances from the cameras in Location A and Location B to ensure matched retinal input from the hand holding the rod. After the ‘*Room*’ phase, a dark curtain was rapidly lowered to cover the visual field for 8 s (‘*Curtain’* phase). During this period, the hand movements were still visible in the foreground. At the end of the ‘*Curtain*’ phase, the experimenter displayed a sign instructing the participants to close their eyes and not open them until feeling the next touch (‘*Eyes closed*’ phase; 2.5 s).

The videos were presented through a pair of MRI-compatible HMDs (Nordic Neurolab, Bergen, Norway) positioned in front of the participant’s eyes and controlled through in-house software. The video recording from the right and left camera were presented in the right and left eye of the HMDs, respectively, resulting in a true stereoscopic video.

### Tactile Stimuli

During the scanning procedures, the experimenter applied tactile stimulation to the participants’ chests. The experimenter stood to the left of the participant and applied the touches using a small rod (the same one used in the videos) attached to the tip of a 1-m wooden stick.

### Experimental Conditions and Design

We employed a 2 × 2 factorial design with the main factors visuo-tactile stimulation mode (SYNCHRONOUS, ASYNCHRONOUS) and location (LOCATION A, LOCATION B). In the SYNCHRONOUS illusion condition, the visual and tactile stimuli were temporally congruent. The same audio cues used during the video recording session were provided to the experimenter to ensure accurate synchronization with the visual stimuli presented to the participant. In the ASYNCHRONOUS control condition, the visual and tactile stimuli were temporally incongruent: the audio commands were delayed for 1 s, resulting in a 1-s delay in the tactile stimuli with respect to the visual stimuli. For every trial, the experimenter was blind as to whether the current condition was SYNCHRONOUS or ASYNCHRONOUS. For both conditions, the view alternated between LOCATION A and LOCATION B. Blocks of all four conditions were presented consecutively in a balanced, pseudo-randomized order. Each condition was repeated 10 times per run, and we collected five runs per participant.

### Post-Scan Behavioral Experiment

**FIGURE 3:**
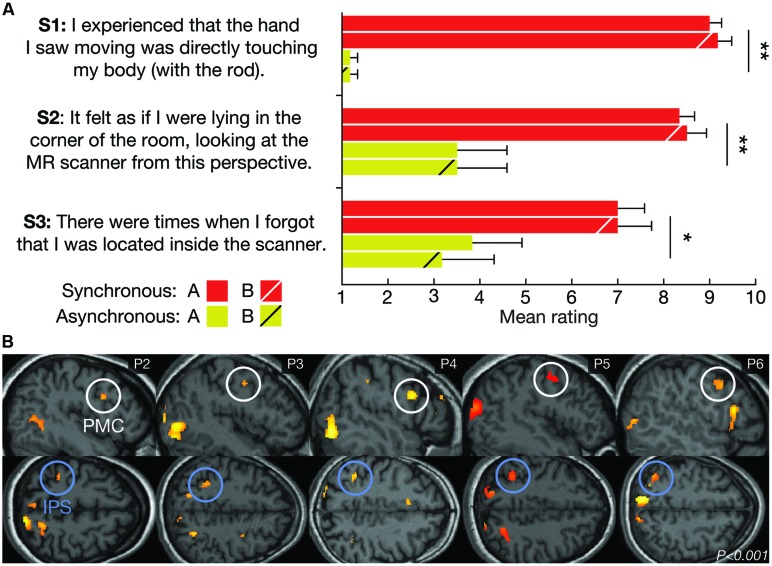
**Subjective and neurophysiological evidence for successful illusion induction. (A)** The average subjective ratings of questionnaire statements S1–S3 for LOCATION A and LOCATION B during the SYNCHRONOUS and ASYNCHRONOUS conditions, respectively. The SYNCHRONOUS condition generated significantly higher ratings for the illusion statements (S1–S3), compared with the ASYNCHRONOUS condition. There was no significant difference in the illusion strength between LOCATION A and LOCATION B. The error bars represent the SEM, and the statistical results refer to the main effect of the visuo-tactile stimulation mode in a 2 × 2 analysis of variance (ANOVA). *P < 0.05, **P < 0.01. **(B)** Examining the main effect of the visuo-tactile stimulation mode using standard univariate analysis revealed increased activity in key multisensory regions in five out of six participants (P2–P6) at the statistical threshold of *P* < 0.001 (uncorrected). One representative brain slice for each subject is shown for the activations in the premotor (PMC, white circles) and intraparietal cortices (IPS, blue circles), which have previously been associated with limb (Ehrsson et al., 2004) and full-body ownership (Petkova et al., 2011; Guterstam et al., 2015b).

We used questionnaires to quantify the subjective experience associated with the illusion (Botvinick and Cohen, 1998; Ehrsson, 2007; Guterstam et al., 2011, 2015a). At the end of the fMRI data acquisition, the participants were requested to remain inside the scanner for an additional couple of minutes, and they were presented with one repetition of each experimental condition. At the end of each block, six different written statements were displayed on the HMDs. Statements 1–3 (see **Figure 3A**) were designed to examine the experience of the illusion. Statements 4–6 (S4: “*When I saw the hand (holding the rod) moving, I experienced touch on my back*,” S5: “*It felt as if I were floating around in the ceiling of the room, looking at the MR scanner from above*” and S6: “*There were times when I forgot who I am.*”) served to control for suggestibility and task compliance. The participants were asked to report verbally the degree of agreement with each statement on a 10-point scale ranging from 1 (“*I do not agree at all*.”) to 10 (“*I agree completely*.”).

To ensure that the participants could clearly discriminate LOCATION A and LOCATION B during the illusion, they were subsequently presented with these two conditions again. After being taken out of the magnet, they were asked to indicate on a map their perceived self-location during the two trials. The map was a proportional map of the room, including the different walls and key landmarks. All of the participants placed their bodies on one of the two beds located in the corners of the room and accurately discriminated LOCATION A and LOCATION B during the SYNCHRONOUS condition.

Finally, we examined whether the appearance of the curtain affected the strength of the illusion. After scanning, we asked the participants to rate the following statement: “*When the curtain came down, I experienced that the vividness of the feeling of being located in the corner of the room*” using an 11-point scale ranging from –5 (“*Strongly decreased*”) to +5 (“*Strongly increased*”) and 0 indicated “*Remained unchanged.*” The illusion decreased slightly in strength (mean rating ± SD: –0.67 ± 0.82), but this effect was not significantly different from 0 (*P* = 0.102, one-sample *t*-test).

The Shapiro–Wilk test was used to examine the parametric assumptions of the data. For normally distributed data sets, we used *t*-tests to analyze the differences between the two conditions and analysis of variance (ANOVA) for differences between more than two conditions. For data sets that were not normally distributed, we used nonparametric Wilcoxon signed-rank tests. Two-tailed tests were used for all of the analyses, and alpha was set to 5%.

### Acquisition of Functional Imaging Data

We acquired high-resolution (voxel size 1.5 mm × 1.5 mm × 1.8 mm) T2*-weighted echo-planar images with blood oxygen level-dependent (BOLD)-contrast (Logothetis et al., 2001) using a Siemens TIM Trio 3T scanner equipped with a 12-channel phased-array head coil. Each functional volume comprised 54 continuous near-axial slices (128 × 124 matrix, TE = 30 ms). This size ensured that the hippocampus was within the FOV, as well as the ventral premotor cortex and (the major part of) the intraparietal sulcus. The cerebellum, the most superior portion of the fronto-parital cortex, and the most caudal portion of the primary visual cortex were outside the FOV. One complete volume was collected every 4 s (TR = 4000 ms). A total of 1250 functional volumes were collected for each participant (250 per run for a total of five runs per experiment). A high-resolution structural image was acquired for each participant at the end of the experiment (3D MPRAGE sequence, voxel size 1 mm^3^, FOV 250 mm × 250 mm, 176 slices, TR = 1900 ms, TE = 2.27 ms, flip angle = 9°). Due to a technical issue with the visual displays, the first run of Participant 1 was discarded.

### Hippocampus Segmentation

An expert (author LB), blinded to the identities of the participants, manually segmented the hippocampi of each participant. The segmentation was performed according to the protocol of Pruessner et al. (2000) with the interactive software package DISPLAY developed at the Brain Imaging Center of the Montreal Neurological Institute (MNI). The protocol includes the fimbria, alveus, dentate gyrus, and cornu ammonis, and it excludes the subiculum.

### Preprocessing

The fMRI data were analyzed with SPM8 (Wellcome Department of Cognitive Neurology, London, UK). The first three volumes of each run were discarded from further analysis due to non-steady-state magnetization. Following slice timing correction, the functional images were realigned to correct for head movements and co-registered with the high-resolution structural scan of each participant. The anatomical image was subsequently segmented into white matter, gray matter, and cerebrospinal fluid partitions. The functional volumes were finally spatially smoothed (see sections below) and analyzed in native space.

### Multivoxel Pattern Analysis

We employed MVPA to examine whether the illusion induced place-specific hippocampal response patterns. In this analysis, the functional data were spatially smoothed using a 3-mm full-width-at-half-maximum (FWHM) isotropic Gaussian kernel in SPM8. Further MVPA-specific preprocessing was performed with the Princeton MVPA toolbox (www.pni.princeton.edu/mvpa) in MATLAB (Mathworks, Natick, MA, USA). BOLD data were extracted for the individually defined hippocampus region-of-interest (ROI). Each voxel’s response was normalized relative to the average of the time course within each scan. The trial labels were shifted by two volumes to account for hemodynamic delay, and linear trends were removed. Single-trial hippocampal responses were formed by averaging across the 8 s that corresponded to the ‘*Curtain*’ phase. Note that this period represents identical visual input that is rigorously controlled for systematic differences among all of the conditions (in particular, between LOCATION A and LOCATION B and between SYNCHRONOUS and ASYNCHRONOUS).

We used linear support vector machines (SVMs; in the LIBSVM implementation, with the fixed regularization parameter of *C* = 1) to compute decoding accuracies. To ensure independent training and testing data, we used a leave-one-run-out cross-validation approach. In this approach, the SVM classifier was initially trained to differentiate between the hippocampal responses to LOCATION A and LOCATION B based on the trials in all but one of the runs; after this training, the classifier was applied to identify the trials of the left-out run as either LOCATION A or LOCATION B. This process was repeated such that each run was left out once, and a final decoding accuracy, representing the run-average percentage of trials that were correctly identified as LOCATION A or LOCATION B, was obtained.

To identify multivoxel patterns, we used locally multivariate mapping (Björnsdotter et al., 2011). The brain was partitioned into regional, overlapping voxel clusters (each of which was approximately spherical in shape and 33 voxels in size); in each of these clusters, a decoding accuracy was computed through the approach that is described above. For each voxel, a representative accuracy was then obtained by computing the mean of the decoding accuracies of the clusters that were associated with the voxel in question. The resulting map reflects robust voxelwise contributions to decoding performance, expressed as the average percentage of correctly identified trials.

Nonparametric permutation testing was used to compute significance levels and conservatively control for familywise error (Nichols and Holmes, 2002; Nichols and Hayasaka, 2003). The identical mapping procedure was iterated 10,000 times with permuted trial labels to generate a probability distribution under the null hypothesis that there was no information regarding the locations (LOCATION A vs. LOCATION B) encoded in the hippocampus ROI. All of the analyses were based on the entire hippocampus ROI (i.e., the left and right hippocampi combined). To correct for familywise error, we identified the maximum map value (i.e., the value of the peak voxel, which represented the mean of the decoding accuracies of all of the clusters that were associated with that voxel, as described in the previous paragraph) for both the true labels and for each of the permutations (Nichols and Hayasaka, 2003; **Figures 2A,C**). A *P*-value was then computed as (1+the number of permuted max values > true max value)/(1+the total number of permutations). To descriptively indicate informative voxels, the same permutation test was applied at the voxel level; in this analysis, *P*-values for each voxel *i* were computed as (1+the number of permuted values_i_ > true value_i_)/(1+the total number of permutations). In this instance, we used a threshold of *P* < 0.001, uncorrected, in accordance with convention in the neuroimaging field (**Figure 2B**; Friston et al., 1995).

First, we examined the hippocampal response patterns during the SYNCHRONOUS condition. Second, we determined whether these multivoxel patterns were specific to the SYNCHRONOUS condition. If the place-specific hippocampal responses are driven by factors unrelated to the illusion (e.g., memory traces of different visual scenes), then the classifier that has been trained on the SYNCHRONOUS trials should decode the ASYNCHRONOUS trials equally well (given that the SYNCHRONOUS and ASYNCHRONOUS trials differ only with respect to the temporal synchrony of the visual and tactile stimuli). However, if the place-specific hippocampal patterns are contingent on the illusion, then the classifier should fail to decode ASYNCHRONOUS trials. To test this issue, we used the classifiers that were trained on the SYNCHRONOUS trials in the leave-one-run-out cross-validation and applied them to the corresponding ASYNCHRONOUS trials (i.e., the corresponding runs that were left out). The results of are presented in **Figure 2**. In addition to the hippocampus, we repeated all analyses in two control regions: the third ventricle and a cortical control area consisting of the frontal eye fields (FEF). The third ventricle ROI was defined using WFU PickAtlas. The bilateral FEF ROI was defined by creating two 8-mm-radius spheres that were centered on the average stereotaxic coordinates of the right and left FEF, respectively (Paus, 1996), and inclusively masked with a gray matter mask.

### General Linear Modeling

For general linear modeling (GLM), the functional data were spatially smoothed using an 8-mm FWHM isotropic Gaussian kernel. GLM analysis was performed with SPM8. For each individual dataset, we fitted a linear regression model to the data. Individual regressors were defined to model the *‘Room’* and ‘*Curtain’* phases separately for the two spatial locations (LOCATION A, LOCATION B) and the visuo-tactile stimulation modes (SYNCHRONOUS, ASYNCHRONOUS), yielding a total of eight separate regressors. One regressor of no interest was defined to model the ‘*Eyes closed*’ phase for all four conditions. Linear contrasts were defined to test for the effects of interest. We examined the contrast SYNCHRONOUS vs. ASYNCHRONOUS irrespective of the location (i.e., main effect of visuo-tactile stimulation mode) and visual input (i.e., during the ‘*Room*’ and ‘*Curtain’* phase combined) to reveal areas modulated by the illusion of owning a full-body (Petkova et al., 2011; Guterstam et al., 2015b). Because of the limited number of participants in the current study, we only studied single-subject activation maps (at the conventional statistical threshold of *P* < 0.001; see **Figure 3B**) and did not perform a group-level random effects analysis. We thus report these results in a purely descriptive fashion.

## Results

We used MVPA (Hassabis et al., 2009; Björnsdotter et al., 2011) to decode perceived self-location from the BOLD response across voxels in the hippocampus. Given the assumption that the out-of-body illusion induces location-specific patterns of activity in hippocampal neuronal populations (Agarwal et al., 2014), this analysis should reveal different place-representations through the detection of subtle variations in the BOLD signal pattern. We exclusively analyzed the period when the dark curtain was presented and performed a control analysis on the asynchronous condition, which was perfectly matched in terms of visual input, to ensure that the internal representation of self-location constituted the key difference between the four experimental conditions. In addition, we repeated the decoding analyses in two control regions outside the hippocampus ROI. To ensure independent model validation, we used a leave-one-run-out approach.

The results showed that perceived self-location could be decoded with remarkable consistency: hippocampal activity patterns significantly distinguished the locations in all six participants in the synchronous condition (*P* < 0.05, corrected, permutation test with 10,000 iterations; **Figures 2A,C**). Voxels informative about self-location were found throughout the hippocampus (**Figure 2B**; **Table 1**). At the group-level, there were no significant differences among the hippocampal subregions (right anterior, right posterior, left anterior, left posterior; χ^2^(3) = 1.316, *P* = 0.725, Freidman test) in the number of informative voxels. All six participants showed significant decoding in the left hippocampus (**Table 1**), which is consistent with the results of recently published study (Guterstam et al., 2015b). To control for factors unrelated to the illusion *per se*, we applied the same multivoxel models to the corresponding asynchronous trials. The decoding was non-significant in all participants in the asynchronous condition (*P* > 0.05, corrected; **Figures 2A–C**). At the group-level, the peak decoding accuracy was significantly lower in the asynchronous compared to the synchronous condition (*t* = 3.87, *P* = 0.012, paired two-tailed *t*-test; **Figure 2A**). These results suggest that the place-specific activity patterns observed in the synchronous condition were not driven by illusion non-specific effects such as differences in visual input.

**Table 1:** Decoding results.

	Cluster size	Peak decoding (%)	MNI			Hc subregion
			*x*	*y*	*z*	
P1	1	62	-18	-19	-17.6	LA
	7	64.25	-31.5	-22	-10.4	LP
	4	66	-34.5	-20.5	-12.2	LA
	1	62.75	-21	-17.5	-14	LA
	5	63.75	31.5	-23.5	-10.4	RP
	1	61	-24	-43	0.4	LP
P2	14	62.2	-27	-13	-24.8	LA
	15	63	22.5	-14.5	-15.8	RA
	15	64	-22.5	-19	-17.6	LA
	1	60.8	-18	-19	-17.6	LA
	7	63	34.5	-35.5	-6.8	RP
	1	59.6	22.5	-40	-1.4	RP
P3	35	66.2	-21	-16	-17.6	LA
	1	61.75	-30	-16	-14	LA
	4	61.2	-22.5	-34	-1.4	LP
P4	5	62.2	-34.5	-11.5	-19.4	LA
	34	67.2	-33	-22	-12.2	LA
	1	60	25.5	-25	-10.4	RP
	3	61.4	30	-37	-3.2	RP
	1	61.4	-16.5	-37	-1.4	LP
	1	59.6	31.5	-34	-1.4	RP
	15	63.8	24	-38.5	2.2	RP
P5	1	61.2	25.5	-17.5	-17.6	RA
	10	64	22.5	-17.5	-14	RA
	1	60.4	-18	-37	-1.4	LP
	1	61	-21	-35.5	-1.4	LP
P6	15	64.25	22.5	-19	-19.4	RA
	1	60.6	36	-14.5	-19.4	RA
	14	65	37.5	-19	-15.8	RA
	1	61	-24	-16	-17.6	LA
	1	61.8	16.5	-37	4	RP

*All of the significant (*P* < 0.001 uncorrected) decoding peaks for each individual participant P1–P6. Hc, hippocampus; LA, left anterior; LP, left posterior; RA, right anterior; RP, right posterior.*

We repeated the decoding analyses in two control regions: the third ventricle and a cortical control area (the FEF, see Materials and Methods). The decoding accuracy in the ventricle was non-significant in all participants for both the synchronous and asynchronous conditions (*P* > 0.05, corrected), suggesting that the hippocampal decoding results cannot be explained by general confounding factors such as condition-related head-movements. The decoding accuracy in the FEF was non-significant for both the synchronous and asynchronous conditions in five out of six participants. In one participant (P1), the FEF decoding accuracy was significant in both the synchronous and asynchronous conditions (*P* < 0.05). These results imply that it is highly unlikely that general visuo-spatial cognitive processes (e.g., covert eye movements) could explain the illusion-specific decoding results in the hippocampus.

Post-scan questionnaires confirmed that all participants vividly experienced the illusion during the synchronous, but not asynchronous, condition (**Figure 3A**). This result is consistent with previous studies (Ehrsson, 2007; Guterstam and Ehrsson, 2012). Contrasting the fMRI data from the synchronous and asynchronous conditions across locations using standard univariate GLM analysis revealed significant activations in the premotor cortex and the intraparietal sulcus in five out of six participants (**Figure 3B**). Activity in these multisensory regions have previously been associated with single-limb (Ehrsson et al., 2004; Gentile et al., 2013; Guterstam et al., 2013) and full-body ownership (Petkova et al., 2011; Gentile et al., 2015; Guterstam et al., 2015b). Although the sample size is small and the results cannot be generalized to the population, these findings provide further support successful illusion induction.

## Discussion

Our results demonstrate an association between hippocampal activity and the perceived location of the bodily self in space. In contrast to previous neuroimaging studies that examined changes in the first-person visual perspective (1PP) (Corradi-Dell’Acqua et al., 2008; Vass and Epstein, 2013), mental imagery of being somewhere else (Lambrey et al., 2012; Marchette et al., 2014), and virtual navigation to target locations (Hassabis et al., 2009; Rodriguez, 2010), the use of the out-of-body illusion allowed us to specifically manipulate the feeling of bodily presence in a given spatial location. We propose that the activity observed in premotor-intraparietal areas constructing multisensory representations of the body (Petkova et al., 2011; Guterstam et al., 2015b) is combined with information from the visual dorsal stream concerning the spatial orientation of environmental landmarks with respect to the first-person visual perspective through the HMDs (Burgess et al., 2002; Burgess, 2006; Yoder et al., 2011). This process could, in turn, provide the hippocampus with information concerning bodily self-location through projections along the parieto-medial temporal pathway (Kravitz et al., 2011) and thereby induce the place-specific hippocampal activation patterns detected in this study.

We suggest that population activity of hippocampal place cells form the likely neuronal substrate for the observed multivoxel patterns. It is well established the activity of individual place cells represent the animal’s position within the local environment (O’Keefe and Nadel, 1978; Moser et al., 2008). However, for MVPA in fMRI to be meaningful, the large-scale spatial distribution of place cells must exhibit sufficient anisotropy to generate BOLD-signal differences across voxels. The proposed existence of such a large-scale hippocampal population code has been controversial (Hassabis et al., 2009; Dombeck et al., 2010). In a recent study, however, Agarwal et al. (2014) showed that a rat’s position in a maze can be decoded from spatially distributed local field potentials in the hippocampus, speaking in favor of an anisotropic nature of the hippocampal population code and the feasibility of our MVPA approach. Earlier fMRI studies on spatial navigation and path integration have demonstrated that the target locations in virtual navigation tasks can be decoded from hippocampal activity patterns (Hassabis et al., 2009; Rodriguez, 2010), which is compatible with the present results. Our findings extend the understanding of the role of the hippocampus in spatial cognition by showing that hippocampal activity patterns reflect perceived bodily self-location and that multisensory integrative mechanisms related to body ownership can update this representation even in the absence path integration.

In a recently published study, we showed that the hippocampus is part of a larger network that includes areas of the posterior parietal and posterior cingulate cortices that work in concert to represent perceived self-location (Guterstam et al., 2015b). In the present study, we used a high-resolution, narrow FOV fMRI protocol to study the hippocampus at a spatial resolution comparable to that of a previous relevant MVPA study focusing on this structure (Hassabis et al., 2009). This fMRI approach came at the expense of major portions of the brain falling outside the FOV (see Materials and Methods), which prevented us from carrying out whole-brain analyses. Nevertheless, the reproducibility of the hippocampal decoding results in each of the six individual subjects, in conjunction with the results of the control analyses performed in the third ventricle and FEF, speak in favor of a high degree of specificity of the hippocampus findings. The observation that self-location could be decoded from the left hippocampus in all participants is compatible with the results of our recently published study, which showed group-level significant decoding of self-location in the left hippocampus (Guterstam et al., 2015b). Together, these results provide strong support for the notion that the left hippocampus is involved in shaping the perceptual experience of self-location.

## Conclusion

Our findings suggest that the human hippocampus is involved not only in spatial navigation (Maguire et al., 1998; Burgess et al., 2002; Hassabis et al., 2009), memory (Nyberg et al., 1996; Squire et al., 2004; Chadwick et al., 2010) and imagining the future (Buckner and Carroll, 2007; Schacter et al., 2012) but also in the perceptual experience of self-location. This observation has bearings on contemporary models of hippocampal function (Maguire and Mullally, 2013; Eichenbaum and Cohen, 2014; Hartley et al., 2014) because it suggests a dynamic interplay between multisensory own-body representations and hippocampal spatial processing.

## Conflict of Interest Statement

The authors declare that the research was conducted in the absence of any commercial or financial relationships that could be construed as a potential conflict of interest.
